# Association between body roundness index and frailty in older Americans: a cross-sectional study of NHANES 2007–2018

**DOI:** 10.3389/fnut.2025.1534464

**Published:** 2025-03-10

**Authors:** Jie Xu, Min Sun, Meng Chen, Zan Lin, Yong Hu, Xiaobing Luo

**Affiliations:** ^1^Department of Sports Medicine, Sichuan Provincial Orthopedics Hospital, Chengdu, China; ^2^Department of Knee Sports Injury, Sichuan Provincial Orthopedics Hospital, Chengdu, China; ^3^Department of Emergency Medicine, Nanchong Hospital of Traditional Chinese Medicine, Nanchong, China

**Keywords:** body roundness index, frailty, obesity, NHANES, cross-sectional study

## Abstract

This study used NHANES data from 2007 to 2018 to examine the relationship between frailty and the Body Roundness Index (BRI) in U.S. people 60 years of age and older. BRI = 364.2–365.5 × sqrt [1–(wc/2π)^2^/(Height/2)^2^]. The degree of frailty was assessed by the frailty index (≥ 0.25). The relationship between frailty and BRI was examined using weighted multivariate logistic regression. To account for potential non-linear patterns, generalized additive modeling (GAM) was utilized, and the ability of BRI to predict frailty was assessed using receiver operating characteristic (ROC) analysis. Results showed that BRI was significantly and positively associated with prevalence of frailty, with a 34% increase in prevalence of frailty per unit increase in a fully adjusted model (OR: 1.34, 95% CI: 1.28, 1.40; *p* < 0.0001). The GAM model showed a significant nonlinear relationship and threshold effect. This study indicates that a higher BRI is closely linked to the onset of frailty in older adults, although additional confirmation through large-scale prospective studies is required.

## 1 Introduction

With the acceleration of global population aging, frailty has become particularly prominent in the elderly population (1). Frailty is a combined state accompanied by the decline of multiple physiological systems that significantly increases the risk of falls, disability, hospitalization, and even death (2). Obesity, particularly abdominal obesity, is widely recognized as one of the main risk factors for frailty. However, the traditional BMI, although widely used in the assessment of obesity (3), is deficient in distinguishing visceral fat from subcutaneous fat, making it difficult to accurately reflect the accumulation of abdominal fat (4), and therefore has limitations in measuring the impact of obesity on frailty. Waist circumference (WC) is a good indicator of abdominal fat accumulation, but it does not provide insight into fat distribution (5). The waist-to-hip ratio provides information on the distribution of abdominal fat, but it does not accurately assess fat metabolism or muscle mass (6). Especially in the elderly population, the lack of age- and gender-specific criteria limits its validity in the prediction of frailty.

The BRI, which combines measurements of height and WC to more accurately capture the features of body fat distribution, particularly the buildup of abdominal fat, was created in order to better evaluate the health risks associated with obesity (7). In contrast to BMI, the BRI has been shown to be more effective in characterizing metabolic syndrome (8), non-alcoholic fatty liver disease (NAFLD) (9), and osteoarthritis (10), among many other health problems, showed higher sensitivity in risk prediction. Additionally, the study found a U-shaped correlation between mortality prevalence and BRI, indicating that both high and low BRI values may elevate mortality rates (11). This finding further underscores the potential of BRI as a valuable tool for health monitoring and prevalence assessment. These studies provide insights into the association of BRI with health outcomes, but BRI is not yet uniformly defined and its clinical application is still under investigation.

While the link between abdominal obesity and frailty has been established in several studies, research examining the relationship between BRI and frailty is still scarce. To address this gap, this study used the National Health and Nutrition Examination Survey (NHANES) data from 2007 to 2018 to investigate the applicability of BRI in older U.S. adults and evaluate its potential role in predicting frailty prevalence. This study aims to enhance our understanding of BRI’s role in frailty screening and provide new support for early intervention in the elderly population.

## 2 Materials and methods

### 2.1 Database sources and sample selection

**Figure 1:**
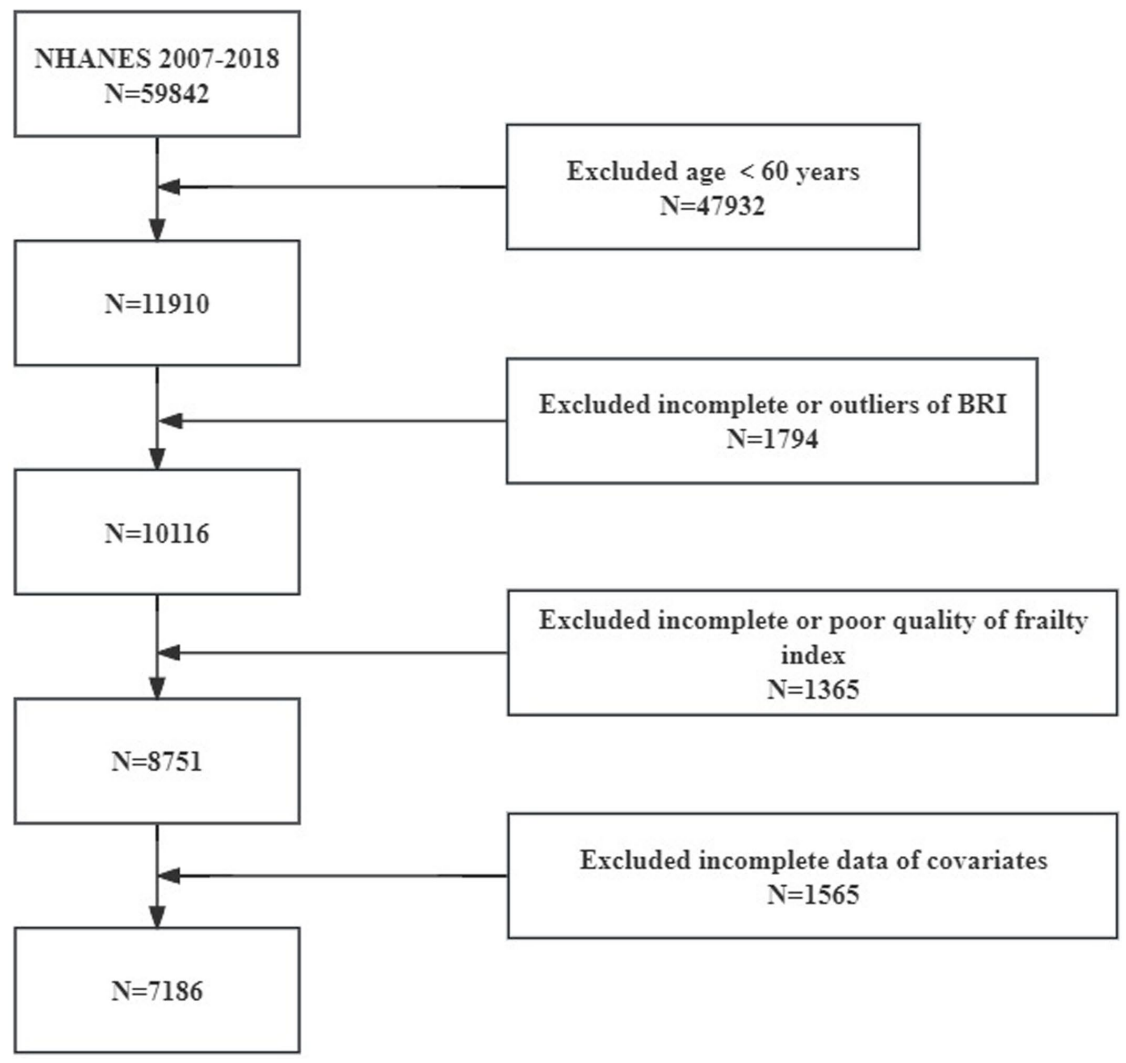
Flowchart of the population selection from NHANES.

The National Centre for Health Statistics (NCHS) administers the NHANES, a nationwide study that evaluates the nutrition and health of American adults living outside of institutions using a stratified multistage sampling method. The data from this survey are accessible to the public, and all participants have given their written consent. Selected data from NHANES between 2007 and 2018 were used for the analyses in this article. Initially, data from 59,842 participants from the NHANES 2007–2018 period were considered. The final sample included 7,186 participants, excluding 47,932 people under the age of 60, 1,794 people with missing or abnormal BRI data, 1,365 people with unreliable frailty index assessments, and 1,565 people with missing covariates (Figure 1). To learn more about NHANES, go to the CDC website at: https://www.cdc.gov/nchs/nhanes/.

### 2.2 Assessment of frailty

Frailty Index (FI) was used to assess frailty, with an FI value ≥0.25 being considered frail. The Frailty Index consists of 49 factors, covering areas such as cognitive function, ability to perform daily activities, physical performance, chronic conditions, overall health, and laboratory test results. Supplementary Table 1 provides the full set of criteria. The severity of each criterion was assessed, with 0 indicating no frailty and 1 indicating severe frailty, and the frailty index was finally obtained by calculating the total score divided by the number of items. To maintain the reliability of the data, only participants who completed 91% or more of the frailty assessment items were considered.

### 2.3 BRI assessment

BRI as an exposure variable was calculated as BRI = 364.2–365.5 × sqrt [1–(wc/2 *π*)^2^/(Height/2)^2^]. All NHANES staff received rigorous training to ensure consistency and accuracy of measurements. To guarantee accuracy, the anthropometric apparatus at every Mobile Examination Centre was regularly calibrated and standardized. BRI data could be analyzed as continuous or categorical variables. BRI values were analyzed by dividing them into three groups (first quartile: 1.12 < BRI ≤ 4.80; second quartile: 4.80 < BRI ≤ 6.37; and third quartile: 6.37 < BRI ≤ 20.15).

### 2.4 Covariates

To account for confounding factors, the study adjusted for several known covariates, including age, gender, race, education level, marital status, poverty income ratio (PIR), alcohol use, smoking, diastolic blood pressure (DBP), systolic blood pressure (SBP), energy intake, and Healthy Eating Index-2015 (HEI-2015). In addition, a history of chronic diseases such as cardiovascular disease, diabetes, chronic kidney disease, and chronic obstructive pulmonary disease, are also potential factors that affect frailty. Specific income levels were categorized as low-income (PIR ≤ 1.3), middle-income (1.3 < PIR ≤ 3.5), and high-income (PIR > 3.5). Smoking was defined as consuming 100 or more cigarettes over one’s lifetime. Alcohol use is classified according to the current drinking status into five categories: never, former, heavy, moderate and mild drinking (12, 13). For detailed classification criteria, see Supplementary Document 2. At least three consecutive standard readings were averaged to estimate blood pressure. On the first day of the 24-h dietary recall study, dietary data were collected. The HEI-2015 evaluates a person’s dietary compliance with the Dietary Guidelines for Americans (14). Higher ratings indicate better food quality and healthier eating habits; the values range from 0 to 100.

### 2.5 Statistical analysis

Every statistical analysis applied the proper sampling weights and considered the intricate sampling design of NHANES. Categorical data are given as weighted proportions, while continuous variables are provided as mean ± standard error (SE). Weighted chi-square and t-tests were used to evaluate group differences at baseline. Model 1 (unadjusted), Model 2 (adjusted for age, gender, race, and education level), and Model 3 (further adjusted for variables such as marital status, PIR, smoking and alcohol consumption, SBP, DBP, HEI-2015, and energy intake) were the three weighted multivariate logistic regression models used to investigate the association between BRI and frailty. GAM were applied to investigate potential nonlinear relationships, while linear regression models were used to examine threshold effects and turning points. Subgroup analyses and interaction tests were performed as well. The effectiveness of the BRI to predict frailty was compared with that of BMI and WC using ROC analysis. DeLong tests were conducted to assess statistical differences in the ROC analysis results. Sensitivity analyses consisted, among other things, of further adjusting for BMI and WC and estimating missing covariates using mean values. A two-tailed *p* < 0.05 was considered statistically significant. All statistical analyses were carried out using EmpowerStates (version 4.2) and R software (version 4.4).

## 3 Results

### 3.1 Baseline characteristics of participants

The general demographics of the 7,186 individuals in the research, whose mean age was 69.43 ± 6.79 years, are shown in Table 1. There were 49.05% females and 50.95% men in the sample, which was very evenly distributed. The mean BRI was 5.82 ± 2. The prevalence of frailty significantly increased as BRI expanded. There were notable variations in the fundamental traits of the various BRI groupings.

**Table 1:** The clinical characteristics of participants.

Characteristics	Total (*N* = 7,186)	Tertile 1 (*N* = 2,395)	Tertile 2 (*N* = 2,391)	Tertile 3 (*N* = 2,400)	*p*-value
		1.12 < BRI ≤ 4.80	4.80 < BRI ≤ 6.37	6.37 < BRI ≤ 20.15	
Age (years)	69.43 ± 6.79	69.54 ± 6.98	69.89 ± 6.82	68.87 ± 6.52	<0.001
Gender %					<0.001
Female	3,525 (49.05)	1,062 (44.34)	1,062 (44.42)	1,401 (58.38)	
Male	3,661 (50.95)	1,333 (55.66)	1,329 (55.58)	999 (41.62)	
Race %					<0.001
Mexican American	805 (11.20)	165 (6.89)	301 (12.59)	339 (14.12)	
Other Hispanic	741 (10.31)	188 (7.85)	272 (11.38)	281 (11.71)	
Non-Hispanic White	3,684 (51.27)	1,232 (51.44)	1,229 (51.40)	1,223 (50.96)	
Non-Hispanic Black	1,452 (20.21)	538 (22.46)	444 (18.57)	470 (19.58)	
Other race	504 (7.01)	272 (11.36)	145 (6.06)	87 (3.62)	
Education level %					<0.001
Less than 9th grade	917 (12.76)	245 (10.23)	313 (13.09)	359 (14.96)	
9-11th grade	962 (13.39)	284 (11.86)	323 (13.51)	355 (14.79)	
High school graduate	1704 (23.71)	537 (22.42)	577 (24.13)	590 (24.58)	
Some college or AA degree	1993 (27.73)	652 (27.22)	640 (26.77)	701 (29.21)	
College graduate or above	1,610 (22.40)	677 (28.27)	538 (22.50)	395 (16.46)	
Marry %					<0.001
Married/Living with partner	4,320 (60.12)	1,471 (61.42)	1,501 (62.78)	1,348 (56.17)	
Widowed/Divorced/Separated	2,507 (34.89)	791 (33.03)	793 (33.17)	923 (38.46)	
Never married	359 (5.00)	133 (5.55)	97 (4.06)	129 (5.38)	
PIR %					<0.001
Low income	1908 (26.55)	585 (24.43)	608 (25.43)	715 (29.79)	
Med income	3,002 (41.78)	967 (40.38)	994 (41.57)	1,041 (43.38)	
High income	2,276 (31.67)	843 (35.20)	789 (33.00)	644 (26.83)	
Alcohol use %					<0.001
Never	2,178 (30.31)	683 (28.52)	702 (29.36)	793 (33.04)	
Former	616 (8.57)	170 (7.10)	200 (8.36)	246 (10.25)	
Mild	3,217 (44.77)	1,139 (47.56)	1,086 (45.42)	992 (41.33)	
Moderate	672 (9.35)	250 (10.44)	211 (8.82)	211 (8.79)	
Heavy	503 (7.00)	153 (6.39)	192 (8.03)	158 (6.58)	
Smoking %					0.208
No	3,475 (48.36)	1,173 (48.98)	1,121 (46.88)	1,181 (49.21)	
Yes	3,711 (51.64)	1,222 (51.02)	1,270 (53.12)	1,219 (50.79)	
BMI (Kg/m^2^)	29.16 ± 6.06	23.74 ± 2.78	28.50 ± 2.58	35.21 ± 5.41	<0.001
WC (cm)	102.53 ± 14.56	88.75 ± 8.24	102.07 ± 6.88	116.75 ± 11.32	<0.001
SBP (mmHg)	133.40 ± 19.70	134.14 ± 20.81	133.09 ± 19.34	132.95 ± 18.90	0.072
DBP (mmHg)	67.65 ± 14.03	68.56 ± 14.23	67.28 ± 14.00	67.10 ± 13.83	<0.001
HEI-2015	54.53 ± 11.41	55.94 ± 11.75	54.88 ± 11.22	52.77 ± 11.03	<0.001
Energy (kcal)	1862.77 ± 779.58	1913.68 ± 797.86	1856.00 ± 761.88	1818.71 ± 775.93	<0.001
Frailty %					<0.001
No	4,887 (68.01)	1897 (79.21)	1715 (71.73)	1,275 (53.12)	
Yes	2,299 (31.99)	498 (20.79)	676 (28.27)	1,125 (46.88)	

Continuous variables were summarized using means with SE, and categorical variables were presented as proportions with SE. GED, general educational development; BMI, body mass index; WC, waist circumference; SBP, systolic blood pressure; DBP, diastolic blood pressure; BRI: body roundness index, PIR: income to poverty ratio.

### 3.2 Multivariate regression analysis

The findings of the weighted multivariate logistic regression analysis are summarized in Table 2. In model 1 (unadjusted), the prevalence of frailty increased 1.36 times for every unit rise in BRI (OR: 1.36, 95% CI: 1.30, 1.41; *p* < 0.0001); in model 2 (adjusted for age, sex, race, and education), the OR was 1.37 (95% CI: 1.31, 1.43; *p* < 0.0001). In fully adjusted model 3, the prevalence of frailty increased by 34% per 1-unit increase in BRI (OR: 1.34, 95% CI: 1.28, 1.40; *p* < 0.0001). Furthermore, Table 2 shows that the highest BRI group had a substantially higher prevalence of frailty than the lowest BRI group when BRI was classified (OR = 3.49, 95% CI: 2.83–4.30; *p* < 0.0001).

**Table 2:** Association between BRI and frailty.

	OR^a^ (95% CI^b^) *P*-value		
	Model 1^c^	Model 1^d^	Model 1^e^
Continuous	1.36 (1.30, 1.41)	1.37 (1.31, 1.43)	1.34 (1.28, 1.40)
	<0.0001	<0.0001	<0.0001
Categories			
Tertile 1	Reference	Reference	Reference
Tertile 2	1.59 (1.33, 1.89)	1.54 (1.28, 1.84)	1.50 (1.24, 1.82)
	<0.0001	<0.0001	<0.001
Tertile 3	3.80 (3.11, 4.64)	3.81 (3.10, 4.67)	3.49 (2.83, 4.30)
	<0.0001	<0.0001	<0.0001
P for trend	<0.0001	<0.0001	<0.0001

OR^a^, odds ratio; 95% CI^b^, 95% confidence interval; Model1^c^, adjusted for non-covariates; Model2^d^, adjusted for age, gender, race, and education; Model3^e^, further adjusted for marry, poverty income ratio, smoking, alcohol use, systolic blood pressure, diastolic blood pressure, healthy eating index-2015 and energy intake.

### 3.3 Nonlinear analysis

**Figure 2:**
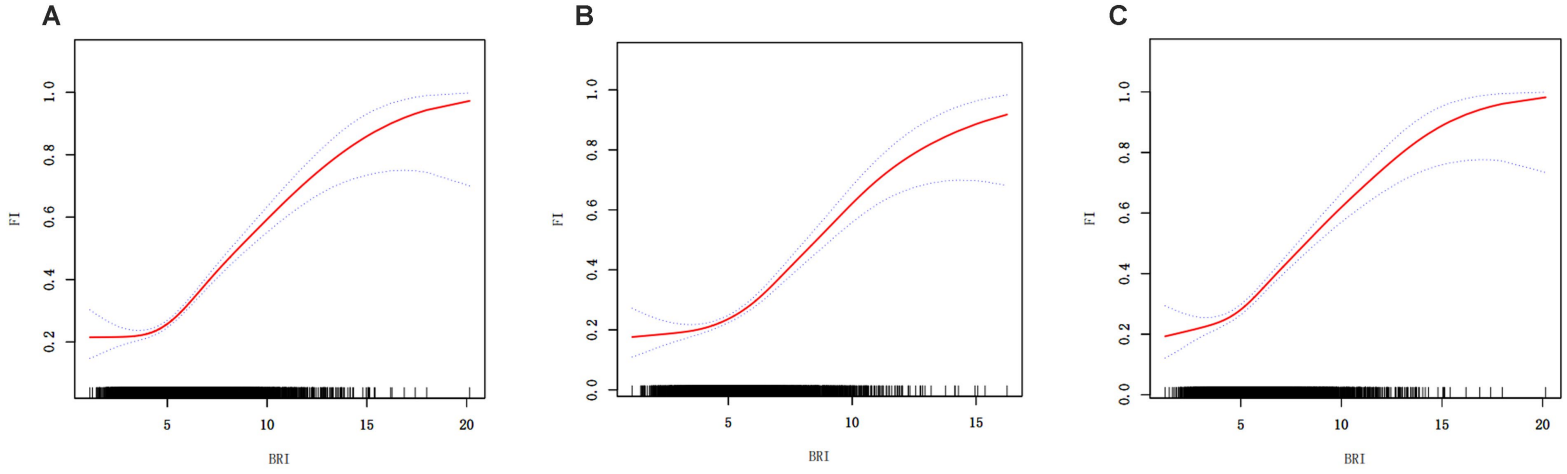
Generalized additive regression. **(A)** GAM for total population; **(B)** GAM for males; **(C)** GAM for females.

GAM was used to further investigate the link between BRI and frailty, and the results showed a significant nonlinear relationship (Figure 2 and Table 3). Segmented regression analysis provided additional evidence supporting the presence of a distinct threshold effect. The nonlinear effect and threshold effect were more significant in males compared to females.

**Table 3:** Segmented regression results.

Gender	OR^a^ (95%CI^b^) *P*-value		
	Total	Males	Females
Segmented model			
Turning point (K)	4.42	4.41	4.54
< K OR 1	0.98 (0.86, 1.11)	0.96 (0.80, 1.15)	1.04 (0.87, 1.24)
	0.7261	0.6503	0.6699
> K OR 2	1.37 (1.32, 1.42)	1.40 (1.33, 1.48)	1.35 (1.29, 1.42)
	<0.0001	<0.0001	<0.0001
OR 2–1	1.40 (1.21, 1.62)	1.46 (1.19, 1.79)	1.30 (1.07, 1.59)
	<0.0001	0.0002	0.0096
Likelihood ratio test	<0.001	<0.001	0.011

OR^a^, odds ratio; 95% CI^b^, 95% confidence interval.

### 3.4 Subgroup analysis

To examine the relationship in different populations, subgroup analyses were conducted, taking into account variables such as age, gender, race, education, marital status, PIR, HEI-2015, BMI, smoking, and alcohol use. The analysis results indicated that the link between BRI and frailty was statistically significant in most subgroups, with particularly strong associations observed in relation to age, race, education, marital status, BMI, and alcohol use. However, differences between gender, PIR, HEI-2015, smoking, and alcohol use groups were not statistically significant (Table 4).

**Table 4:** Results of subgroup regression.

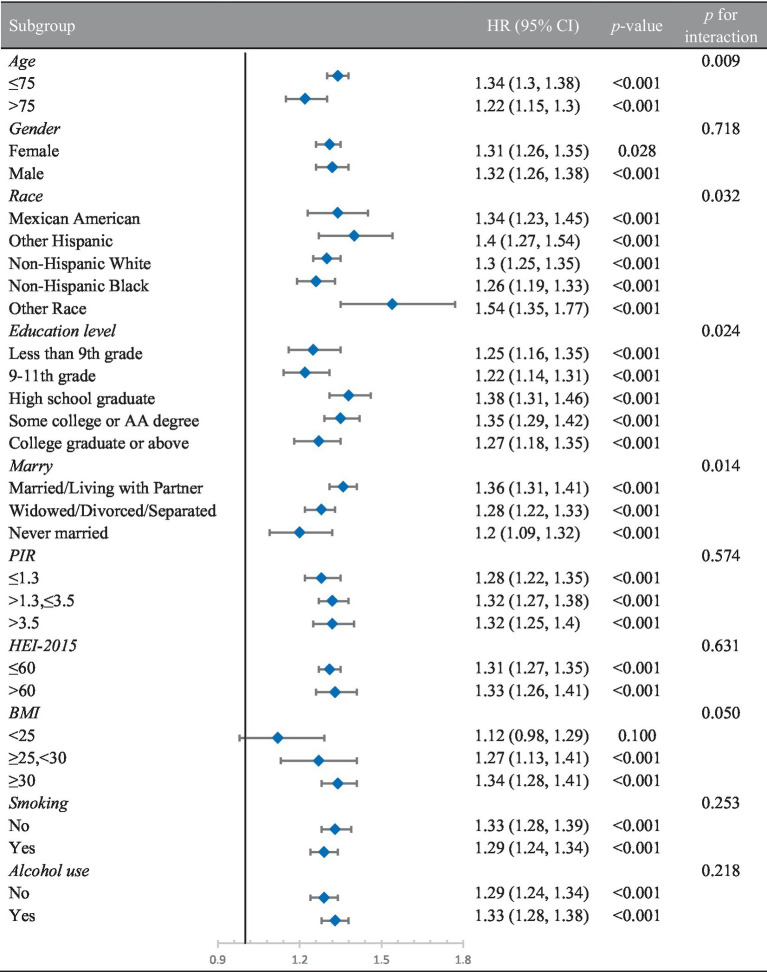

Results of the subgroup analysis were adjusted for all covariates except the effect modifier.

### 3.5 Sensitivity analysis

Several sensitivity analysis were conducted to make sure the results were reliable. These included imputations for missing variables and adjustments for conditions including cardiovascular disease, diabetes, chronic kidney disease, chronic obstructive pulmonary disease, and the use of antihyperlipidemic and antidiabetic drugs. The results showed high stability, with the sensitivity analyses repeatedly confirming a substantial link between frailty and BRI (Table 5).

**Table 5:** Further adjustment for disease, and medication conditions.

	OR^a^ (95% CI^b^) *P*-value	
	Model 4^c^	Model 5^d^
Continuous	1.31 (1.23, 1.40)	1.29 (1.12, 1.48)
	< 0.001	0.003
Categories		
Tertile 1	Reference	Reference
Tertile 2	1.44 (1.05, 1.98)	1.11 (0.68, 1.81)
	0.035	0.668
Tertile 3	3.27 (2.31, 4.64)	3.19 (1.81, 5.62)
	< 0.001	0.002
P for trend	< 0.001	< 0.001

OR^a^, odds ratio; 95% CI^b^ 95% confidence interval; Model 4^c^, further adjustment for diabetes, CVD, COPD, CKD; Model 5^d^, further adjustment for the use of antidiabetic, and antihyperlipidemic drugs.

### 3.6 ROC analysis

**Figure 3:**
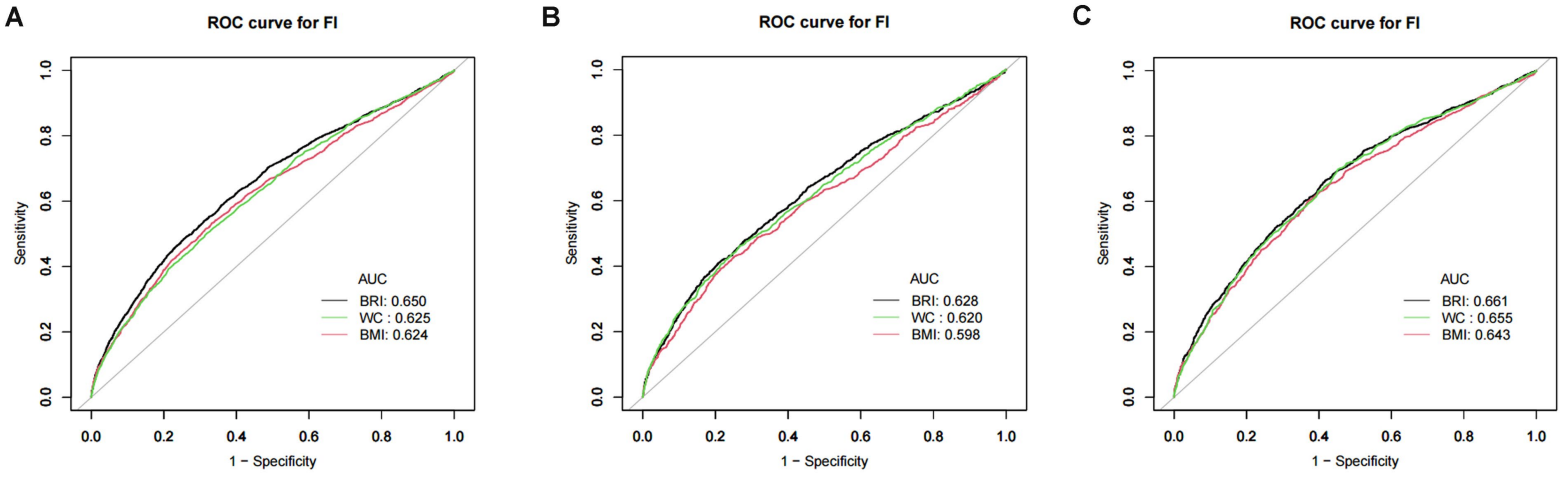
ROC curve of BRI, BMI, and WC. **(A)** ROC for total population; **(B)** ROC for males; **(C)** ROC for females. Note: BRI, body roundness index; BMI, body mass index; WC, waist circumference.

The predictive ability of BRI, BMI, and WC for frailty was assessed by ROC analysis (Figure 3). The findings demonstrated that BRI outperformed BMI and WC as a predictor of frailty across all populations, with statistically significant differences (Table 6).

**Table 6:** ROC analysis results.

	Variable	AUC (95% CI)	Threshold	Sensitivity	Specificity	Youden index	*P*-value
Total	BRI	0.65 (0.64, 0.66)	6.41	0.75	0.48	0.23	-
	BMI	0.62 (0.61, 0.64)	29.85	0.67	0.53	0.2	<0.001
	WC	0.63 (0.61, 0.64)	106.15	0.68	0.50	0.18	<0.001
Males	BRI	0.63 (0.61, 0.65)	6.41	0.79	0.41	0.2	-
	BMI	0.60 (0.58, 0.62)	30.81	0.75	0.43	0.18	<0.001
	WC	0.62 (0.60, 0.64)	110.25	0.74	0.46	0.2	0.026
Females	BRI	0.66 (0.64, 0.68)	5.82	0.59	0.66	0.25	-
	BMI	0.64 (0.62, 0.66)	29.41	0.63	0.60	0.23	<0.001
	WC	0.65 (0.64, 0.67)	97.55	0.55	0.70	0.25	0.049

BRI, body mass index; BMI, body mass index; WC, waist circumference.

## 4 Discussion

Using NHANES data from 2007 to 2018, this study examined the relationship between BRI and the prevalence of frailty in U.S. persons 60 years of age and older. The findings showed that BRI and a higher prevalence of frailty were significantly and independently correlated. A 34% increase in the prevalence of frailty was associated with every unit rise in BRI in the fully adjusted model. GAM analysis further highlighted a nonlinear relationship, showing a 37% increase in frailty prevalence for each unit rise in BRI when BRI exceeded 4.42. The results of the ROC curve analysis indicated that BRI outperformed BMI and WC in the prediction of frailty prevalence. The AUC values of BRI were significantly higher than those of BMI and WC (*p* < 0.05), further demonstrating the superiority of BRI in distinguishing high-risk populations. In addition, to enhance the robustness of the results, sensitivity analysis, and subgroup analysis were performed in this study. After adjusting for BMI and WC, the association between BRI and frailty prevalence remained significant, indicating strong stability of the study results. In subgroup analyses, significant associations between BRI and frailty were observed in groups of different age, gender, race, smoking, and drinking statuses. This suggests that the BRI has good applicability and predictive power for a wide range of older populations.

Research on frailty has extensively explored the effects of obesity, BMI, and metabolic health states (15). The study by He et al. (16) states that metabolic status plays a key role in the progression of frailty, and metabolically unhealthy obesity (MUOO) significantly accelerated the progression of frailty, while metabolically healthy obesity (MHOO) had a lesser effect on frailty (16). Several studies have revealed that BRI, an important indicator of body fat distribution, is strongly associated with a variety of health problems. For instance, research by Zhang et al. (17) identified a significant association between BRI and the prevalence of non-alcoholic fatty liver disease (NAFLD), highlighting a non-linear relationship. The study by Li et al. (8) noted that BRI is a significant predictor of metabolic syndrome, and its nonlinear characterization suggests that individuals with high BRI have a significantly increased prevalence of metabolic disorders. BRI has been linked to a higher prevalence of depression among U.S. adults (18). In addition, the BRI has shown superiority in predicting the prevalence of osteoporosis and osteoarthritis (10, 19). It should be noted that the systematic review and meta-analysis by Yuan et al. (3) showed that abdominal obesity was significantly associated with the risk of frailty (RR = 1.57). The study also found that both excessively high and low BMI levels were associated with higher frailty rates, indicating a U-shaped relationship between BMI and frailty. Meanwhile, Zhang et al. (11) demonstrated a U-shaped relationship between BRI and adult mortality in the United States, suggesting that either too low or too high a BRI value may be associated with increased mortality. Although our study focused only on the positive correlation between BRI and frailty, this result should not be interpreted to mean that only higher BRI is associated with frailty. Possible reasons for this include the fact that this study included only the elderly population in the United States, of which only 79 had a BMI below the normal range, a relatively small sample size, which limited our ability to effectively assess the potential association between low BRI and frailty. Low BRI may be associated with low body fat or insufficient fat reserves, which is also a risk factor for frailty (20). Therefore, future studies should be conducted in larger sample sizes of older adults and in different populations. While prior research has examined the connection between BMI, abdominal obesity, and frailty, studies specifically investigating the link between BRI and frailty remain limited. The present study reveals for the first time the potential of BRI, an emerging metric, in the prediction of frailty prevalence, especially in revealing the nonlinear characteristics and threshold effects of BRI. This study aligns with the findings of Yuan et al. (3) and He et al. (16) in supporting the positive correlation between obesity and frailty prevalence. However, the present study used BRI instead of BMI or WC as a measure of obesity, which better reflects the comprehensive nature of body fat distribution.

Obesity increases the prevalence of frailty through multiple pathophysiologic processes. First, abdominal fat is a metabolically active tissue that secretes a variety of pro-inflammatory factors (e.g., tumor necrosis factor alpha, interleukin-6), leading to systemic chronic low-grade inflammation (21). This inflammatory state is thought to be one of the main drivers of frailty, as inflammation not only accelerates the loss of muscle mass and strength but may also accelerate multisystem decline through oxidative stress (22). Individuals with a high BRI are typically accompanied by higher abdominal fat stores and are thus more susceptible to the negative effects of inflammation. It may also give rise to sarcopenic obesity, a coexisting state of excess adipose tissue, and deficient muscle mass, which is often closely associated with elevated BRI (23). In this state, muscle is replaced by adipose tissue, leading to decreased muscle function and strength, as well as reduced metabolic efficiency. This dual effect of muscle deficiency and obesity makes the probability of weakness significantly higher. Second, the accumulation of abdominal fat is directly related to the development of insulin resistance, the latter of which can further lead to metabolic disorders (e.g., diabetes, hyperlipidemia, etc.) (24). Metabolic disorders may accelerate the onset of frailty through a number of pathways, e.g., affecting muscle glucose metabolism capacity (25) decreasing the rate of protein synthesis (26), and impaired muscle repair (27). Additionally, there is a substantial correlation between on the prevalence of cardiovascular disease, a significant risk factor for frailty, and abdominal obesity, and a higher BRI may further accelerate the progression of frailty through damage to the cardiovascular system. Specific mechanisms include increased atherosclerosis, decreased cardiac output, and deterioration of the heart’s pumping function (28). All of these factors impair physical performance and mobility in older adults. At the same time, abdominal obesity is associated with several hormonal imbalances, including leptin (29), insulin-like growth factor (IGF-1) (30), and abnormalities in sex hormone binding globulin (SHBG) (31). These hormones are closely related not only to energy metabolism and fat distribution but also have important effects on muscle mass and bone strength. Individuals with high BRI tend to exhibit higher leptin resistance and low levels of IGF-1 (32) which may further exacerbate the frail state. Finally, excessive obesity is usually accompanied by higher levels of psychological problems such as depression and anxiety (33), and these factors may further accelerate the onset of frailty through indirect effects such as reduced physical activity levels, altered dietary habits, and exacerbation of chronic inflammation. In addition, the present study observed a threshold effect between BRI and frailty, which may reflect the body’s ability to adapt to fat accumulation within a certain range. When BRI is low, the body may maintain homeostasis through metabolic compensation mechanisms, but when BRI exceeds a certain level, the metabolic burden increases rapidly, leading to increased inflammatory responses and metabolic disorders, thus significantly increasing the prevalence of frailty.

This study’s use of NHANES data, which offers a sizable, nationally representative sample and improves the findings’ generalisability and dependability, is one of its main strengths. The study adjusted for a variety of potential confounders, including age, gender, race, and lifestyle factors, enhancing the reliability of the findings. Subgroup analyses confirmed a significant association between BRI and frailty across various populations, reinforcing the robustness of the findings. However, the study also had certain limitations. A causal association between BRI and frailty could not be demonstrated since the data came from a cross-sectional survey; more longitudinal research is required to confirm this. Despite adjusting for multiple variables, there may be potential confounders (e.g., genes, etc.) that were not captred. The study population was an older U.S. population, and the applicability of the results to other regions or ethnicities requires further validation.

## 5 Conclusion

According to this study, BRI and frailty among US older persons are significantly positively correlated. Compared to standard BMI and WC, the BRI offers a higher predictive potential for frailty. These results highlight BRI’s promise as a screening and management tool for frailty, but larger prospective trials are required for additional validation.

## Data Availability

Publicly available datasets were analyzed in this study. This data can be found at: https://www.cdc.gov/nchs/nhanes/.
